# Surgery

**Published:** 1878-11

**Authors:** 


### Surgery.

### Reuniting a Severed Sciatic Nerve.

In his address before the Surgical Section of the British Med. Association, Mr. Wheelhouse, of Leeds, related the case of a man who had fallen upon a scythe, and completely divided the sciatic nerve, the injury leading to paralysis of the limb, which resisted all treatment. An attempt was made to re-unite the ends by exposing them by careful dissection, and paring them obliquely, until apparently fresh nerve tissue was exposed. They were then brought together, and stitched with fine carbolized catgut thread. The leg had to be firmly flexed upon the thigh, in order to make up the shrinkage in the nerve length, and the ankle was firmly lashed to the buttock to retain it in this position.

Gradually sensation returned. At the end of five weeks he relaxed the position of the leg, letting it down inch by inch, till it was straight again. Slowly the power of voluntary motion also returned, and three months after his admission to hospital, he was discharged able to walk with the aid of two canes. Improvement continued, and eventually he was able to work and move about as he was wont to do.

Myositis Ossificans.—(*Med. Press and Circular*, August 21, 1878.)

At a recent meeting of the Vienna Medical Society, Dr. Nicoladoni presented a girl, seven years old, as an example of that very rare affection of the muscles, *ossification.* The disease had been going on about a year, commencing in the muscles of the neck, whence it extended to the spine, the anterior part of the thorax and the limbs.

On each side of the spine a rigid line (sacro-spinalis) extends. The scapula is fixed to the thorax; and in the cervical regions are found fibrous cords containing bony plates. The right knee-joint is contracted, and the pectorales are almost entirely ossified. There are but few similar cases on record.

